# Viral infection/reactivation during long-term follow-up in multiple myeloma patients with anti-BCMA CAR therapy

**DOI:** 10.1038/s41408-021-00563-8

**Published:** 2021-10-18

**Authors:** Di Wang, Xia Mao, Yimei Que, Menglei Xu, Yuhang Cheng, Liang Huang, Jue Wang, Yi Xiao, Wen Wang, Guang Hu, Shangkun Zhang, Tongcun Zhang, Chunrui Li, Jianfeng Zhou

**Affiliations:** 1 Department of Hematology, Tongji Hospital of Tongji Medical College, Huazhong University of Science and Technology, Wuhan, Hubei 430030 China; 2 Immunotherapy Research Center for Hematologic Diseases of Hubei Province, Wuhan, Hubei 430030 China; 3 Nanjing IASO Biotherapeutics Ltd, Nanjing, Jiangsu 210032 China; 4 College of Life Science and Health, Wuhan University of Science and Technology, Wuhan, Hubei China; 5 Wuhan Bio‐Raid Biotechnology Co., Ltd., Wuhan, Hubei China

**Keywords:** Infectious diseases, Immunotherapy

**To the Editor:**

Targeting B-cell maturation antigen (BCMA) through monoclonal antibody or chimeric antigen receptor (CAR) T-cell therapies have achieved encouraging results in treating refractory/relapsed multiple myeloma (MM) patients [1–5]. These BCMA-targeting strategies eliminate not only tumor cells but also parts of normal plasma and B cells that express BCMA [6]. These on-target off-tumor effects may lead to severe deficiency of humoral immunity and make patients susceptible to infections, which has already raised concern in CD19-CAR-treated patients [7, 8]. As we have conducted two anti-BCMA CAR trials in our center, we did observe a remarkably higher incidence of viral infection/reactivation during the long-term follow-up. To date, few studies have investigated the influence of anti-BCMA immunotherapies on viral infection/reactivation. This retrospective study focuses on the viral infection in patients treated with anti-BCMA CAR, aiming to provide feasible measures to manage viral infection/reactivation in patients receiving BCMA-targeting immunotherapy.

Participants were patients from two different anti-BCMA CAR T-cell trials, registered on Chinese Clinical Trial Registry (http://www.chictr.org.cn) with registry numbers ChiCTR-OPC-16009113 and ChiCTR1800018137, for murine- and fully human-originate CAR, respectively. Four patients participated in both trials and were identified as independent subjects in either trial [4, 5]. Patients who died or lost to follow-up within 1 month post infusion were excluded (Fig. S1). Finally, from January 2017 to September 2019, 39 patients were enrolled from the murine-originate CAR trial and 22 patients were enrolled from the fully human-originate CAR trial. All the patients had received Epstein-Barr virus (EBV), cytomegalovirus (CMV), hepatitis B/C virus (HBV/HCV), and human immunodeficiency virus (HIV) tests at screening and serial points post infusion, which were not predesignated in the protocol. Except for chronic HBV, no prophylaxis was given post infusion. Medical records for all patients were reviewed for demographic, clinical, treatment, and survival information. The cut-off date was 31 January 2021.

The study protocols were approved by the Institutional Review Board of Tongji Hospital, Tongji Medical College, Huazhong University of Science and Technology. Written informed consent was obtained from each participant in compliance with the Declaration of Helsinki. Fisher’s exact test was employed to analyze the categorical variables. The estimation of the probabilities of overall survival (OS) was performed by the Kaplan-Meier method. The statistical analyses were performed by SPSS 22 and GraphPad Prism 8. *P* values less than 0.05 (two-tailed) were considered statistically significant.

The general characteristics of the participants are shown in Table S1. The patients had a median age of 55.0 (range, 34.0–70.0) years and a median time from diagnosis of 37.0 (range, 8.0–151.0) months. Of the patients, 41.0% had a high-risk cytogenetic profile, and 41.0% had extra-medullary myeloma or plasma cell leukemia. The median line of therapies before enrollment was 4 (range, 3–11). Viral infection/reactivation events were recorded separately before and after infusion, as summarized in Fig. 1A. There were ten viral DNA replication events in nine patients recorded before infusion. In the meantime, a total of 18 viral infection/reactivation events were recorded in 15 patients after infusion, including four EBV, six CMV, three HBV, four herpes zoster, and one COVID-19. The rate of viral infection/reactivation observed post infusion was significantly higher than that in published studies involving myeloma patients with chemotherapy [9, 10], indicating that anti-BCMA CAR can negatively affect the anti-viral immunity.

**Fig. 1:**
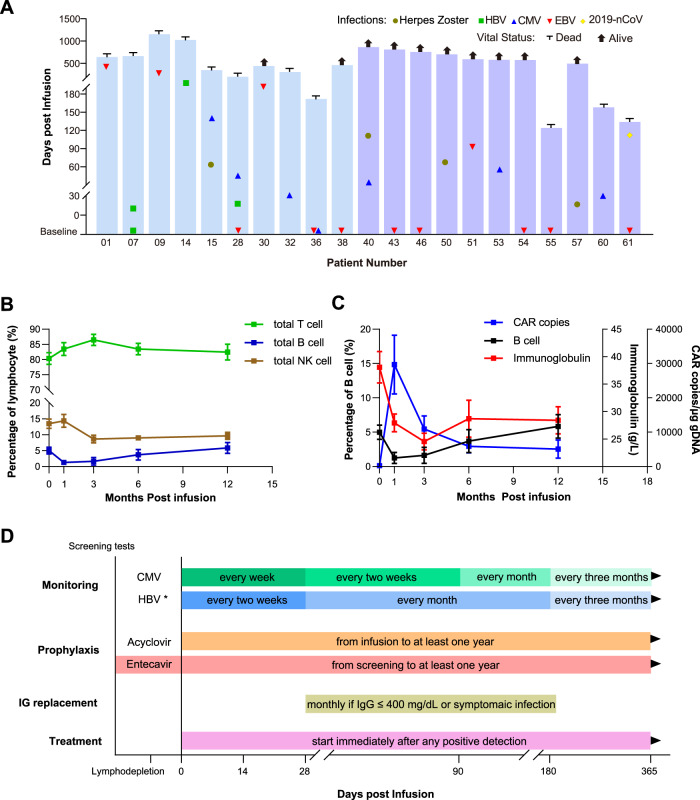
Viral infections, changes of immune system, and recommendations of viral management in patients receiving BCMA-targeting CAR T-cell therapy. **A** All infection events detected from screening till the data cut-off date or last follow-up are shown; each symbol represents a virus type. The blue and purple bars represent the overall survival of patients receiving mouse- and fully human-originate anti-BCMA CAR, respectively. The viral DNA replication detected at screening was marked on the baseline, including one HBV, one CMV, and eight EBV cases. After anti-BCMA CAR T-cell infusion, 18 viral infection/reactivation events were recorded, including four EBV, six CMV, three HBV, four herpes zoster, and one COVID-19 case. Most events were recorded within the first 6 months post infusion. **B** The percentage of lymphocyte subpopulations, including T cells, B cells, and NK cells, was analyzed by flow cytometry. B cells and NK cells fell to the nadir at the first and the third months post infusion, respectively, while T cells peaked at 3 months post infusion. The bar at each point represents the standard error of the mean. **C** BCMA CAR transgene and immunoglobulin levels in patients were tested post infusion. BCMA CAR transgene reached the peak at the first months, which was parallel with the depletion of B cells. Decrease of immunoglobulin fell behind the B cells depletion and recovered from the sixth month. The bar at each point represents the standard error of the mean. **D** At the time of patient enrollment, screening tests of common viruses, such as HBV, HCV, HIV, CMV, and EBV, would be needed. Regular monitoring of viral nucleic acid after anti-BCMA CAR infusion is recommended. Long-time entecavir prophylaxis is necessary for patients with HBV infection, and acyclovir prophylaxis is recommended for all patients. Immunoglobulin replacement is preferred for patients with IgG ≤ 400 mg/dL or symptomatic infection. Antiviral treatment would be needed immediately after positive viral findings except for EBV. Abbreviations: CMV, cytomegalovirus; EBV, Epstein-Barr virus; HBV/HCV, hepatitis B/C virus; HIV, human immunodeficiency virus; IG, Immunoglobulin. *Only patients who are confirmed with HBV infection (including chronic and resolved infection) need regular monitoring of viral nucleic acid post infusion.

CMV and EBV are members of the Herpesviridae family and can persist life-long as a latent infection in B cells and monocytes following the acute phase of infection. When the immune response is compromised by immunosuppressive therapies, both CMV and EBV can reactivate and cause clinical disease. One patient had detectable CMV DNA at screening without clinical symptoms and was treated prior to infusion. Six patients had CMV reactivation with a median load of 4.54 × 10^3^ (range, 1.85 × 10^2^–5.06 × 10^4^) copies/mL after anti-BCMA CAR infusion, including three symptomatic and three asymptomatic reactivations (Table S2). All reactivation events occurred in the first 6 months post infusion (Fig. S2), and were treated preemptively with ganciclovir and immunoglobulin. The median time from reactivation diagnosis to CMV DNA negativity in peripheral blood was 26 days (range, 10–57). As shown in Fig. S3, EBV DNA replication was detected in eight patients at baseline with a median load of 1.1 × 10^3^ (range, 6.96 × 10^2^–3.0 × 10^4^) copies/mL. Interestingly, although no anti-EBV intervention was given, all the pre-existing EBV infections were automatically eradicated within 2 months post infusion. This phenomenon is probably because of the elimination of EBV-infected B cells by the on-target off-tumor effect of anti-BCMA CAR. Four patients had EBV reactivation after infusion, which were all asymptomatic. Two reactivation events were detected at the time of relapse or disease progression, one of which was eliminated by following fully human-originate CAR (Fig. S3). None of the four asymptomatic patients received anti-EBV therapy. Notably, more CMV reactivations were observed in patients treated with glucocorticoids, while fewer EBV reactivations were found in glucocorticoid-treated patients (Table S3).

As China is one of the epidemic areas of HBV, effective screening and drug prophylaxis in HBV-infected patients are essential for the participants. At screening, resolved HBV infection was detected in 47.5% (29/61) of the patients. Four patients (6.45%) had chronic HBV infection and were given entecavir as prophylaxis, among which one (patient 07) had active HBV DNA replication (Table S4). HBV reactivation was observed in three patients (Fig. S4), including patient 07, whose HBV DNA copies significantly increased post infusion and peaked at 1.23 × 10^5^ IU/mL at day 49. Another chronic infection (patient 28) had peak DNA copies at the first month post infusion. It is worth noting that one resolved infection (patient 14) who had no prophylaxis treatment encountered reactivation 6 months post infusion, and was finally controlled by entecavir. This case indicates that resolved-infection patients also need drug prophylaxis after anti-BCMA CAR treatment; otherwise, there is a risk of viral reactivation or even fulminant hepatitis.

One patient who achieved stringent complete response was infected by COVID-19 2 months post infusion. The patient was admitted to intensive care unit and received multiple antiviral therapies including umifenovir, lopinavir/ritonavir, and intravenous immunoglobulin. The immune system failed to generate neutralizing antibodies to prevent COVID-19 proliferation. Unfortunately, he passed away 28 days after diagnosis of COVID-19, as described by Wei et al. [11]. Herpes zoster was reported in four patients who did not receive any antiviral prophylaxis after CAR therapy. The onset time varied from 1 to 3 months post infusion, and the patients were cured with antiviral agents such as acyclovir or valaciclovir.

We monitored CAR transgene copy, lymphocyte subset, and serum immunoglobulin level at serial points post infusion. As shown in Fig. 1B, the percentage of total T cells kept increasing for 3 months to the peak, representing the expansion of CAR-T cells. The percentage of B cells and NK cells tended to decline post infusion, and the nadir of B cells occurred at 1 month. Till the data cut-off, B-cell recovery was observed only in 25 patients, with a median time of 4 (range, 1–10) months (Fig. S5). We also monitored the CAR transgene copy. The peak arose within 1 month, which was parallel with the nadir of B cells. Meanwhile, the decrease of immunoglobulin lagged behind the B-cell depletion. The minimum of immunoglobulin level emerged at the third month post infusion, and hypogammaglobulinemia persisted for about 6 months (Fig. 1C). This long-term abnormality in lymphocyte subsets and hypogammaglobulinemia demonstrated a profound negative impact on the immune system by BCMA-targeting.

Several measures can be taken to diminish the adverse effect of BCMA-targeting on viral immunity, as outlined in Fig. 1D. Firstly, a thorough screening test for viruses is recommended before lymphodepletion. CAR-T infusion would be postponed until treatment and eradication of any baseline active viral reactivations/infections, except for EBV. Also, viruses such as CMV and HBV need regular monitoring post infusion, since their reactivation may negatively affect survival [12]. Secondly, prophylactic antiviral therapy is essential [8]. Oral antiviral agent, such as acyclovir, is recommended after neutrocyte recovery. For CMV, more aggressive monitoring and prompt treatment after positive detection is recommended, as acyclovir may not be enough. We also suggest that both resolved and chronic HBV infections use anti-HBV drug such as entecavir from screening [13]. Thirdly, according to the hypogammaglobulinemia recovery curve, regular immunoglobulin infusion is preferred [8, 14]. But vaccination, especially attenuated live vaccine, is not recommended in the first 6 months since antibody synthesis is paralyzed [8, 15]. Lastly, reactivations of CMV, HBV, and other symptomatic viral infections detected post infusion would need immediate intervention to reduce the potential negative impact on survival (Fig. S6).

In summary, viral infection/reactivations are common adverse events in patients receiving anti-BCMA CAR therapy because of persistent B-cell aplasia and hypogammaglobulinemia. Therefore, prophylaxis and regular monitoring of the virus are recommended. Since this retrospective study was limited to a relatively small sample size, and some infections might be underreported by the patients during the long-term follow-up, a prospective study of a larger scale is required in the future.

## Supplementary information

Supplemental material

## Data Availability

The datasets used and/or analyzed during the current study are available from the corresponding author on reasonable request.

## References

[1] Brudno JN, Maric I, Hartman SD, Rose JJ, Wang M, Lam N (2018). T cells genetically modified to express an anti-B-cell maturation antigen chimeric antigen receptor cause remissions of poor-prognosis relapsed multiple myeloma. J Clin Oncol.

[2] Raje N, Berdeja J, Lin Y, Siegel D, Jagannath S, Madduri D (2019). Anti-BCMA CAR T-cell therapy bb2121 in relapsed or refractory multiple myeloma. N Engl J Med.

[3] Lonial S, Lee HC, Badros A, Trudel S, Nooka AK, Chari A (2020). Belantamab mafodotin for relapsed or refractory multiple myeloma (DREAMM-2): a two-arm, randomised, open-label, phase 2 study. Lancet Oncol.

[4] Li C, Cao W, Que Y, Wang Q, Xiao Y, Gu C (2021). A phase I study of anti-BCMA CAR T cell therapy in relapsed/refractory multiple myeloma and plasma cell leukemia. Clin Transl Med.

[5] Wang D, Wang J, Hu G, Wang W, Xiao Y, Cai H (2021). A phase 1 study of a novel fully human BCMA-targeting CAR (CT103A) in patients with relapsed/refractory multiple myeloma. Blood.

[6] Novak AJ, Darce JR, Arendt BK, Harder B, Henderson K, Kindsvogel W (2004). Expression of BCMA, TACI, and BAFF-R in multiple myeloma: a mechanism for growth and survival. Blood.

[7] Hill JA, Li D, Hay KA, Green ML, Cherian S, Chen X (2018). Infectious complications of CD19-targeted chimeric antigen receptor-modified T-cell immunotherapy. Blood.

[8] Hill JA, Seo SK (2020). How I prevent infections in patients receiving CD19-targeted chimeric antigen receptor T cells for B-cell malignancies. Blood.

[9] Nucci M, Anaissie E (2009). Infections in patients with multiple myeloma in the era of high-dose therapy and novel agents. Clin Infect Dis.

[10] Blimark C, Holmberg E, Mellqvist UH, Landgren O, Björkholm M, Hultcrantz M (2015). Multiple myeloma and infections: a population-based study on 9253 multiple myeloma patients. Haematologica.

[11] Wei J, Zhao J, Han M, Meng F, Zhou J (2020). SARS-CoV-2 infection in immunocompromised patients: humoral versus cell-mediated immunity. J Immunother Cancer.

[12] Teira P, Battiwalla M, Ramanathan M, Barrett AJ, Ahn KW, Chen M (2016). Early cytomegalovirus reactivation remains associated with increased transplant-related mortality in the current era: a CIBMTR analysis. Blood.

[13] Cao W, Wei J, Wang N, Xu H, Xiao M, Huang L (2020). Entecavir prophylaxis for hepatitis B virus reactivation in patients with CAR T-cell therapy. Blood.

[14] Hill JA, Giralt S, Torgerson TR, Lazarus HM (2019). CAR-T – and a side order of IgG, to go? – Immunoglobulin replacement in patients receiving CAR-T cell therapy. Blood Rev.

[15] Walti CS, Krantz EM, Maalouf J, Boonyaratanakornkit J, Keane-Candib J, Joncas-Schronce L (2021). Antibodies to vaccine preventable infections after CAR-T-cell therapy for B-cell malignancies. JCI Insight.

