# Supplementary material for: Viral infection/reactivation during long-term follow-up in multiple myeloma patients with anti-BCMA CAR therapy
Source: Blood Cancer J. 2021 Oct 18;11(10):168. doi: 10.1038/s41408-021-00563-8 (PMC8521092; doi:10.1038/s41408-021-00563-8)
Supplement: Supplementary file 1 — Supplemental material [file 41408_2021_563_MOESM1_ESM.docx]

**Supplements**

This Supplemental material is provided by the authors for additional information about their work.

Supplements to Di Wang*, et al.* **Viral infection/reactivation during long-term follow-up in Multiple Myeloma Patients with anti-BCMA CAR therapy**

**CONTENTS:**

**Supplemental Tables**

Table S1. Basic characteristics of all 18 patients at screening

Table S2. Clinical manifestations and treatment of CMV reactivations post infusion

Table S3 Clinical characteristics related with viral infection after BCMA CAR T cell therapy

Table S4. Status of HBV infection at screening

**Supplemental Figures**

Fig. S1. Flow chat of this trial

Fig. S2. Bubble chart of the time-to-virus load of CMV in patients before and after anti-BCMA CAR infusion

Fig. S3. Bubble chart of the time-to-virus load of EBV in patients before and after anti-BCMA CAR infusion

Fig. S4. HBV DNA copies and liver function of the patient with HBV reactivation

Fig. S5. Time to recovery of B cell.

Fig. S6. Survival analysis of patients with or without viral infection

**Supplementary Tables**

**Table S1.** **Basic characteristics of all 18 patients at screening**

| **Characteristics** | **Mouse sCFV**  **N=39** | **Human sCFV**  **N=22** | **Total**  **N=61** |
| --- | --- | --- | --- |
| Median age (range), y | 55.0 (34 - 70) | 52.5 (38 - 67) | 55.0 (34 - 70) |
| Gender (M/F) | 21/18 | 14/8 | 35/26 |
| Median time since diagnosis^*^ (range), m | 39.0 (8 - 151) | 34.5 (12 - 94) | 37.0 (8 - 151) |
| ISS staging |  |  |  |
| I | 12 | 11 | 23 |
| II | 10 | 10 | 20 |
| III | 13 | 1 | 14 |
| NA | 4 | 0 | 4 |
| High risk cytogenetic features^†^, N | 16 | 9 | 25 |
| EMM and/or PCL | 18 | 7 | 25 |
| Median prior lines of therapy (range) | 4 (3 - 11) | 4 (3 - 6) | 4 (3 - 11) |
| Auto-HSCT, N | 12 | 7 | 19 |
| Best response |  |  |  |
| CR/sCR | 17 | 16 | 33 |
| VGPR | 5 | 4 | 9 |
| PR | 12 | 2 | 14 |
| MR/SD/PD | 5 | 0 | 5 |
| CRS grade |  |  |  |
| Grade 0-2 | 31 | 16 | 47 |
| ≥Grade 3 | 8 | 6 | 14 |
| Glucocorticoid use | 15 | 15 | 30 |
| B cell recovery | 20 | 5 | 25 |

*Defined as the time spanning the initial diagnosis till screening in this study.

†High risk cytogenetic features included following abnormalities detected by conventional cytogenetics or fluorescence in-situ hybridization: del(17p), t(4;14), or t(14;16)

Auto-HSCT: autologous hemopoietic stem cell transplantation; EMM: extra-medullary myeloma; ISS: International Staging System; PCL: plasma cell leukemia

**Table S2. Clinical manifestations and treatment of CMV reactivations post infusion**

| **Patient No.** | **Time of positive detection** | **Clinical symptoms** | **Treatment** | **Time of negative detection** |
| --- | --- | --- | --- | --- |
| 15 | Day 139 | diarrhea | Ganciclovir | Day 165 |
| 28 | Day 43 | No symptom | Ganciclovir, IVIG | Day 69 |
| 32 | Day 29 | fever | Ganciclovir, IVIG | Day 52 |
| 40 | Day 32 | No symptom | Ganciclovir, IVIG | Day 89 |
| 53 | Day 53 | imaging changes of the lung, fever, thrombocytopenia | Ganciclovir, Foscarnet sodium, IVIG | Day 63 |
| 60 | Day 28 | No symptom | Ganciclovir, IVIG | Day 54 |

CMV: cytomegalovirus; No.: number; IVIG: intravenous immunoglobulin

**Table S3. Clinical characteristics related with viral infection after BCMA CAR T cell therapy**

| **Characteristics** | **Total viral infection**  **n=15** | ***p*** | **EBV reactivation**  **n=4** | ***p*** | **CMV reactivation**  **n=6** | ***p*** |
| --- | --- | --- | --- | --- | --- | --- |
| Age > median | 5 | 0.488 | 0 | 0.085 | 2 | 0.688 |
| Gender (M/F) | 9/6 | 0.552 | 3/1 | 0.460 | 4/2 | 0.628 |
| Time since diagnosis > median | 8 | 0.711 | 2 | 0.973 | 3 | 0.966 |
| ISS staging III | 2 | 0.308 | 0 | 0.259 | 1 | 0.700 |
| High risk cytogenetic features | 6 | 0.406 | 2 | 0.704 | 3 | 0.636 |
| EMM and/or PCL | 6 | 0.406 | 1 | 0.501 | 3 | 0.636 |
| Prior lines of therapy > 4 | 6 | 0.715 | 2 | 0.548 | 3 | 0.454 |
| Auto-HSCT | 6 | 0.394 | 2 | 0.400 | 3 | 0.294 |
| Best response ≥ VGPR | 10 | 0.833 | 3 | 0.784 | 5 | 0.420 |
| CRS grade > 2 | 4 | 0.694 | 0 | 0.259 | 2 | 0.524 |
| Glucocorticoid use | 9 | 0.334 | 0 | 0.042^*^ | 6 | 0.009^*^ 0.008* |
| B cell recovery | 4 | 0.194 | 2 | 0.704 | 1 | 0.202 |

Abbreviations: Auto-HSCT, autologous hemopoietic stem cell transplantation; CMV, cytomegalovirus; CRS, cytokine release syndrome; EBV, Epstein-Barr virus; EMM, extra-medullary myeloma; ISS, International Staging System; PCL, plasma cell leukemia

**p* value is statistically significant

**Table S4. Status of HBV infection at screening**

| **Characteristics** | **HBsAg** | **HBcAb** | **HBV DNA** | **n** |
| --- | --- | --- | --- | --- |
| Chronic HBV infection | + | + | + | 1 |
|  |  |  | - | 3 |
| Resolved infection | - | + | - | 29 |
| HBV Negative | - | - | - | 29 |

Abbreviations: HBV, hepatitis virus B; HBsAg, hepatitis B surface antigen; HBcAb, hepatitis B core antibody

**Supplementary Figures**

**Fig. S1.**

**Screened n=29**

**Failed n=3***

**Dosed n=23**

**Enrolled n=22**

**Receive Lymphodepletion n=26**

**Not dosed n=3^†^**

**Excluded n=1^‡^**

**Screened n=44**

**Failed n=3***

**Dosed n=41**

**Enrolled n=39**

**Receive Lymphodepletion n=41**

**Excluded n=2^‡^**

**Observation n=61**

**Fig. S1. Flow chat of this trial**

* Three patients failed in screening of trail ChiCTR-OPC-16009113, and three patients failed in screening ChiCTR1800018137, respectively, because of not meet inclusion criteria or rapid progression

† Three patients who received lymphodepletion were not dosed because of heart failure, severe liver function damage, and severe infection, respectively.

‡ Exclusion criteria was early death or loss of follow-up within one month.

**Fig. S2.**

**
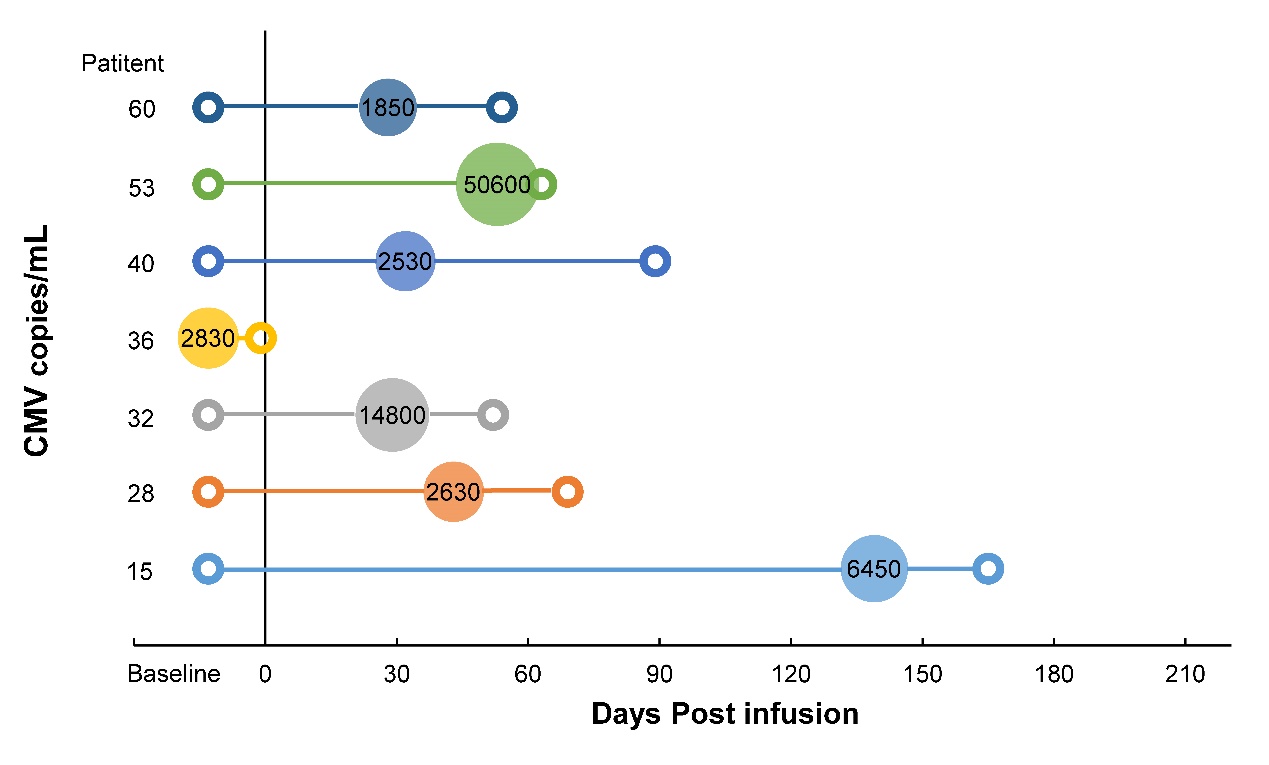
**

**Fig. S2. Bubble chart of the time-to-virus load of CMV in patients before and after BCMA CAR T cell infusion** CMV DNA was monitored by quantitative real-time polymerase chain reaction at screening and serial points post infusion. The diameter of bubbles represents the natural logarithmic of CMV copies. CMV negative was defined as less than 400 copies/mL and was shown as a hollow circle. At baseline, only one patient was detected with CMV DNA replication. After infusion, six patients had no CMV DNA replication at screening. The median CBV copy was 4.54×10^3^ (range, 1.85×10^2^ to 5.06×10^4^) copies/mL for the six patients. The median time from infection diagnosis to CMV DNA negativity in blood was 26 days (range, 10 to 57).

**Fig. S3**

**
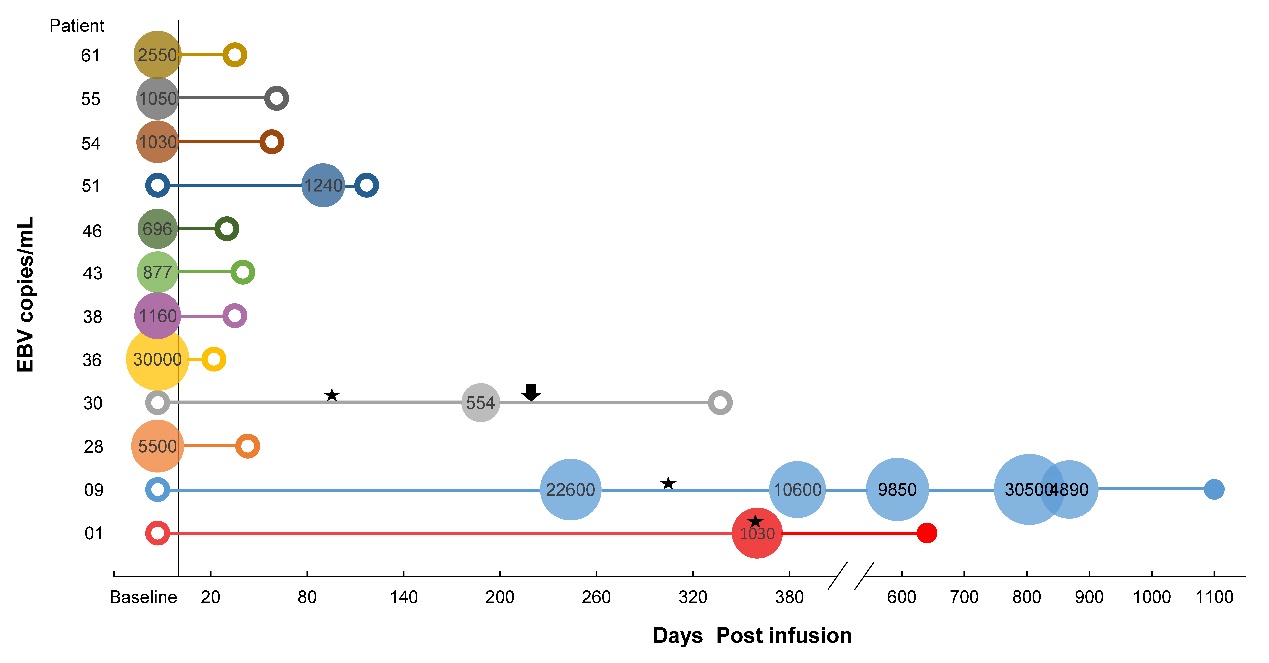
**

**Fig. S3. Bubble chart of the time-to-virus load of EBV in patients before and after BCMA CAR T cell infusion** EBV DNA was monitored by quantitative real-time polymerase chain reaction at screening and at serial points post infusion. The diameter of bubbles represents the natural logarithmic of EBV copies. EBV negative was defined as less than 500 copies/mL and was shown as a hollow circle. At baseline, eight patients were detected with EBV DNA replication. The median EBV copy was 1.1×10^3^ (range, 6.96×10^2^ to 3.0×10^4^) copies/mL. ALL the eight patients had EBV eliminated within two months post infusion. After infusion, four patients who had no EBV DNA replication at screening were detected with EBV reactivation. Patient 01 had relapsed disease when EBV DNA was first detected to be positive. The EBV infection persisted till death, shown in a solid circle. Patient 09 had relapsed disease two months after EBV was first detected. After various strategies, the patient remained relapsed, and the EBV infection persisted till death, shown in a solid circle. Patient 30 had progressive disease at day 96, and then EBV DNA was detected to be positive. He then received the fully human BCMA CAR therapy, and EBV was eliminated. Patient 51 only had a transient EBV reactivation during remission state. The stars represented the time of relapse or progressive disease. The arrow showed the time of fully human BCMA CAR infusion.

**Fig. S4.**

**
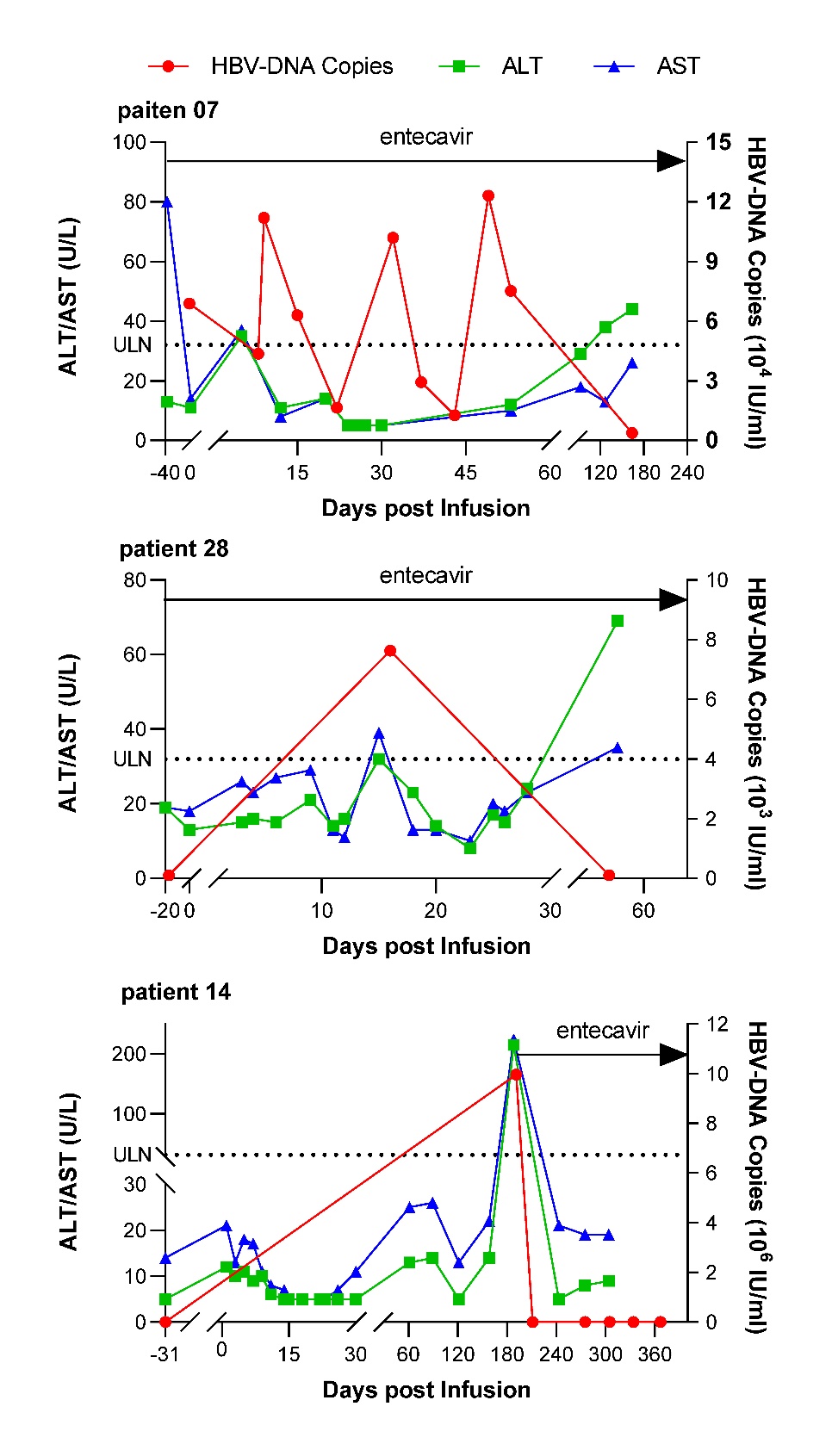
**

**Fig. S4. HBV DNA copies and liver function of the patient with HBV reactivation** Patient 07 and 28 had chronic HBV infection, defined as serologically positive for hepatitis B surface antigen (HBsAg), and was given entecavir as prophylaxis. Patient 07 had a high level of HBV DNA (2.36 × 10^4^ IU/mL) at baseline. The HBV DNA level reached the first peak of 1.12 × 10^5^ IU/mL at day nine post infusion, and then fluctuated. The HBV DNA became undetectable on day 164 post infusion. Serum levels of ALT and AST were always around the normal range. Patient 28 had negative HBV DNA at screening but had HBV reactivation at day 16 post infusion, with a 7.63 × 104 IU/mL peak. Serum levels of ALT and AST continued to increase after HBV reactivation. The HBV DNA became undetectable on day 45 post infusion. Patient 14 was resolved infection, defined as negative HBsAg but positive for antibody against hepatitis B core antigen, and was not given antiviral prophylaxis after infusion. She was detected with HBV reactivation on day 191 post infusion with ALT, and AST levels increased to more than four times the upper limit of normal. Following entecavir treatment, a declined HBV DNA copies and serum levels of ALT and AST were observed. Abbreviations: ALT, alanine aminotransferase; AST, aspartate aminotransferase; HBV, hepatitis B virus; ULN, upper limit of normal.

**Fig. S5**


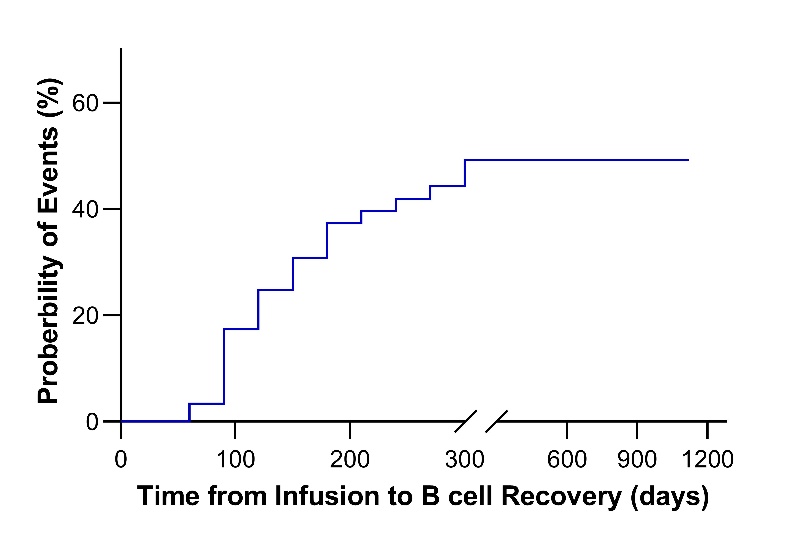


**Fig. S5. Time to recovery of B cell** Time to recovery of B cell (time elapsed from infusion till the first time of recovery, which is defined as absolute CD19-positive or CD20-positive B cell count >20 cells/mm^3^) in patients is shown. The median time for B cell recovery is 4 (range, 1 to 10) months

**Fig. S6**


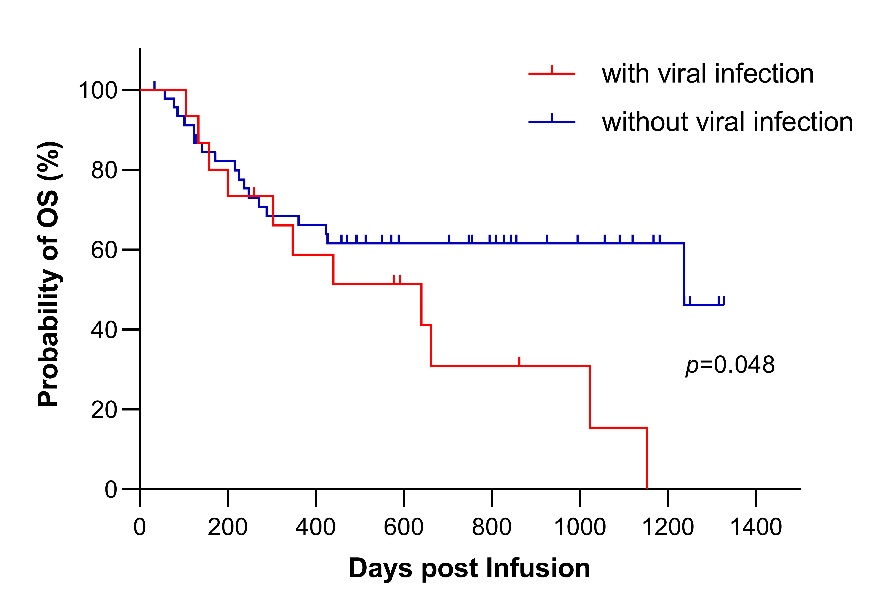


**Fig. S6. Survival analysis of patients with or without viral infection** Overall survival (OS) according to viral infection status was shown. Of the patients with viral infection/reactivations, the median OS was 640 days; without viral infection/reactivations, the median OS was 1238 days. There was a significant difference in OS between the two groups (*P* = 0.048).
